# Ghrelin Protects Against Insulin-Induced Hypoglycemia in a Mouse Model of Type 1 Diabetes Mellitus

**DOI:** 10.3389/fendo.2020.00606

**Published:** 2020-09-11

**Authors:** Kripa Shankar, Deepali Gupta, Bharath K. Mani, Brianna G. Findley, Sherri Osborne-Lawrence, Nathan P. Metzger, Chen Liu, Eric D. Berglund, Jeffrey M. Zigman

**Affiliations:** ^1^Department of Internal Medicine, Center for Hypothalamic Research, UT Southwestern Medical Center, Dallas, TX, United States; ^2^Department of Neuroscience, UT Southwestern Medical Center, Dallas, TX, United States; ^3^Division of Endocrinology, Department of Internal Medicine, UT Southwestern Medical Center, Dallas, TX, United States; ^4^Department of Psychiatry, UT Southwestern Medical Center, Dallas, TX, United States

**Keywords:** ghrelin, type 1 diabetes, glucose clamp, hypoglycemia, glucose counterregulation

## Abstract

Insulin-induced hypoglycemia is a major limiting factor in maintaining optimal blood glucose in patients with type 1 diabetes and advanced type 2 diabetes. Luckily, a counterregulatory response (1) system exists to help minimize and reverse hypoglycemia, although more studies are needed to better characterize its components. Recently, we showed that the hormone ghrelin is permissive for the normal CRR to insulin-induced hypoglycemia when assessed in mice without diabetes. Here, we tested the hypothesis that ghrelin also is protective against insulin-induced hypoglycemia in the streptozotocin (2) mouse model of type 1 diabetes. STZ-treated ghrelin-knockout (KO) (3) mice as well as STZ-treated wild-type (WT) littermates were subjected to a low-dose hyperinsulinemic-hypoglycemic clamp procedure. The STZ-treated ghrelin-KO mice required a much higher glucose infusion rate than the STZ-treated WT mice. Also, the STZ-treated ghrelin-KO mice exhibited attenuated plasma epinephrine and norepinephrine responses to the insulin-induced hypoglycemia. Taken together, our data suggest that without ghrelin, STZ-treated mice modeling type 1 diabetes are unable to mount the usual CRR to insulin-induced hypoglycemia.

## Introduction

Insulin-induced hypoglycemia is prevalent in type 1 diabetes and advanced type 2 diabetes (4). Given the high risk of morbidity and mortality associated with hypoglycemia, mammals have developed a highly integrated counterregulatory response (CRR) system to help prevent, minimize, and reverse hypoglycemia. In individuals without diabetes, this CRR system is mobilized to varying degrees during progression from fasting to starvation. As reviewed by Cryer (5), in humans, the first defense within the traditional CRR involves decreased insulin secretion, which disinhibits glycogenolysis and gluconeogenesis and reduces glucose uptake into muscle and fat. The second defense involves increased glucagon release, stimulating hepatic glycogenolysis and gluconeogenesis. The third defense is an increase in epinephrine, resulting in higher delivery of gluconeogenic substrates to the liver and inhibition of whole body glucose utilization and insulin release. Fourth and fifth defenses include cortisol and growth hormone (GH) rises, which mobilize if hypoglycemia persists and work by limiting glucose utilization and stimulating gluconeogenesis. A sixth defense is activation of the sympathetic nervous system, which, in conjunction with adrenal activation, is linked to a constellation of warning symptoms that include tachycardia, tremors, anxiety, irritability, arousal, sweating, and hunger. Many of these responses are influenced directly or indirectly via glucose-sensing neurons in the central nervous system (6).

Importantly, this CRR system is often compromised in diabetic patients experiencing insulin-induced hypoglycemia (5). For instance, an attenuated sympathoadrenal response may occur, manifesting as hypoglycemia-associated autonomic failure and a markedly increased risk for severe hypoglycemia (7). The normal fine-tuning of insulin release is not an option due to β-cell failure. Also, α-cell dysregulation may occur, due to a lack of functional β-cells in the diabetic islet, and thus loss of normal tonic inhibition of α-cells by intra-islet insulin. This can lead to a reduced glucagon response to insulin-induced hypoglycemia (8).

Although the CRR during insulin-induced hypoglycemia in the context of diabetes has been long appreciated, it is not fully understood and also likely includes other hormones besides those originally described by Cryer. As an example, the stomach-derived hormone ghrelin has many known glucoregulatory actions in both rodent models and humans and seems well-suited to participate in the CRR. Administration of ghrelin (which henceforth mainly refers to the acylated form of ghrelin) reduces insulin sensitivity, restricts insulin secretion, stimulates glucagon secretion and GH release, and raises circulating cortisol (9–12). These interactions, as well as those with GLP-1 (13), and ghrelin receptor (GHSR)-expressing neurons in the hypothalamic arcuate nucleus and caudal brainstem (1, 14), likely contribute to ghrelin's overall glucoregulatory effects, which are emphasized by the actions of administered ghrelin to increase blood glucose (11, 12, 15–20) and conversely, by the blood glucose-lowering effects of ghrelin deletion or blockade, as reviewed in (9, 10).

Regarding the latter, chronic pharmacological blockade of GHSRs or genetic ablation of other ghrelin system components improve glucose tolerance in diet-induced obese mice (21–26). An intact ghrelin system also is required to prevent development of severe hypoglycemia and resulting death in a mouse starvation model. More specifically, ghrelin-KO mice exhibit a progressive decline in fasting blood glucose to the point of near-death following a week-long 60% caloric restriction regimen that depletes body fat to <2% (27). Hypoglycemia under this regimen also occurs in mice with ablated ghrelin cells, mice deficient in ghrelin *O*-acyltransferase, mice overexpressing the endogenous GHSR antagonist LEAP2, GHSR-null mice, and mice with ghrelin cell-selective deletion of β_1_-adrenergic receptors (14, 24, 28–30).

A functional ghrelin system also appears to be important in various diabetic models, as reviewed in (10). As examples, ghrelin deletion markedly attenuates hyperglycemia in leptin-deficient (*ob/ob*) mice, which are hyperphagic, obese, and diabetic (31). GHSR antagonist administration normalizes blood glucose in otherwise hyperglycemic HNF1α-deficient mice – a model of maturity-onset diabetes of the young type 3, which is associated with elevated plasma ghrelin (32, 33). Administration of a GHSR inverse agonist improves glucose tolerance in Zucker diabetic fatty (ZDF) rats (25). Also, type 1 diabetes as modeled in rats and mice by chemical ablation of pancreatic β-cells with streptozotocin (STZ) causes elevation of plasma ghrelin (34–41). Genetic ablation of ghrelin or pharmacological inhibition of GHSR cause significant reductions in STZ-associated hyperphagia (36, 38–40, 42). Furthermore, genetic ablation of GHSR lowers fasting blood glucose in STZ-treated mice (42).

In a recent study, we specifically investigated the contributions of ghrelin to the CRR to insulin-induced hypoglycemia using mice without diabetes (41). We showed that ghrelin-KO mice exhibit more pronounced and prolonged hypoglycemia than WT littermates when administered the same insulin dose in the form of a single bolus (41). Also, ghrelin-KO mice required a much (10-fold) higher glucose infusion rate (GIR) to maintain the same target blood glucose as WT littermates during low-dose hyperinsulinemic-hypoglycemic clamps (41), similar to the findings in GHSR-KO mice (43). Ghrelin-KO mice also exhibited less robust corticosterone and GH responses than their WT counterparts during the clamps (41). Conversely, ghrelin receptor agonist (HM01) administration, which reduced the GIR required by ghrelin-KO mice during the clamps, increased plasma corticosterone and plasma GH (41).

Collectively, these data suggest that endogenously-produced ghrelin not only influences insulin sensitivity, but also is permissive for the normal CRR to insulin-induced hypoglycemia. As the CRR is altered in diabetes (see above), we undertook the current study in order to investigate the protective actions of ghrelin during insulin-induced hypoglycemia in the STZ model of type 1 diabetes.

## Materials and Methods

### Animals

All animal experiments performed in this study were approved by the UT Southwestern Medical Center Institutional Animal Care and Use Committee. Ghrelin-KO mice [line GKO1 (41, 44)] and WT littermates on a C57BL/6N background were generated by pairing mice heterozygous for the ghrelin-KO allele. Ghrelin-KO mice contain two copies of the ghrelin-KO allele whereas WT littermates instead contain two copies of the wild-type ghrelin allele. Mice were housed at room temperature (21.5–22.5°C) using a 12 h light-dark cycle and were provided *ad lib* access to water and regular chow [2016 Teklad Global 16% Protein Rodent Diet (Envigo, Indianapolis, IN)], except as noted.

### Jugular Vein Catheterization

Eight to ten week-old male mice were anesthetized using 2% isoflurane, and each was then surgically implanted with a right jugular vein catheter (0.20-inch, Silastic tubing) (Instech Laboratories, Plymouth Meeting, PA). The free end of the catheter was exteriorized from the dorsal intrascapular region, and the incision sites were closed with a 5–0 nylon suture. Mice were fitted with a vascular harness (Instech Laboratories). Mice were provided ketoprofen (5.25 mg/kg s.c.) immediately, 24, and 48 h post-surgically for analgesia. Mice also were closely monitored post-surgically for signs of infection or swelling, neither of which were observed.

### Administration of STZ

To model type 1 diabetes mellitus, 2 d following jugular vein catheterization, mice were injected once with STZ (150 mg/kg i.p.; Sigma-Aldrich, St. Louis, MO) freshly dissolved in sodium citrate buffer (0.1 M, pH~4.5). Drinking water containing 10% dextrose was provided for 24 h in petri dishes on the floors of the home cages to preclude development of transient hypoglycemia. *Ad lib*-fed blood glucose levels were checked before administration of STZ and 3 d later via tail nick using a Contour Next EZ glucometer system (Ascensia Diabetes Care, Parsippany, NJ). Mice with blood glucoses > 200 mg/dL were used for clamp studies. Only two mice (both of which were ghrelin-KO mice) were excluded from the clamp studies due to day 3 post-STZ injection blood glucoses ≤ 200 mg/dL. Blood glucoses at or above the maximum detection limit (600 mg/dL) were noted as 600 mg/dL for quantification purposes.

### Low-Dose Hyperinsulinemic–Hypoglycemic Clamp Procedure

Hyperinsulinemic-hypoglycemic clamps were performed in conscious, unrestrained mice 4 d after STZ injection as described previously (41), using modifications that took into account their baseline hyperglycemia in the *ad lib*-fed state. Mice were fasted overnight for 16 h before starting the clamp procedure at 10:00 AM (with access to water until 9:00 AM). This 16 h fast preceding the clamp studies was longer than that used previously in non-STZ-treated mice (5 h) (41) as it was needed to achieve the target hypoglycemic range using the low-dose insulin. During the clamp, Humulin-R insulin was infused over 2 h at a constant rate of 4 mU/kg/min i.v. Simultaneously, a solution of 20% glucose (prepared using pharmaceutical grade 50% dextrose diluted in sterile saline) was infused i.v. at a variable rate to achieve and maintain blood glucose levels within a target range of 35–45 mg/dL during the final 20 min of the 2-h clamp procedure. Blood glucose was measured via tail nicks every 5 min, as above. Blood samples to measure ghrelin were taken from tail nicks at *t* = −5 and *t* = 120 min. At *t* = 120 min, blood samples to measure insulin, epinephrine, norepinephrine, glucagon, corticosterone, and GH were collected by cardiac puncture from mice that had been anesthetized with isoflurane.

### Histologic Assessment of Islets

A separate group of ghrelin-KO and WT littermates (*n* = 5–7) received a single i.p. injection of vehicle (0.1 M sodium citrate buffer, pH~4.5) or STZ (150 mg/Kg; freshly prepared, as above). Three days later, blood glucose levels of mice in the *ad lib*-fed state were measured, as above, and blood to assess plasma insulin was collected by quick superficial temporal vein bleed. On the same day, mice were deeply anesthetized with chloral hydrate, transcardially perfused with formalin, and then processed for islet histology. Eight μm-thick pancreatic sections were cut on a cryostat at 50-μm intervals, mounted, and then assessed for both insulin-immunoreactivity (red) and glucagon-immunoreactivity (green). Incubations with primary antibodies and secondary antibodies [guinea pig anti-Insulin (DakoCytomation, Carpinteria, CA; diluted 1:300) followed by Alexa Fluor 594^®^ donkey anti-guinea pig IgG (ThermoFisher Scientific; 1:500); rabbit anti-Glucagon (Millipore, Temecula, CA; diluted 1:300) followed by Alexa Fluor 488^®^ donkey anti-rabbit IgG antibody (ThermoFisher Scientific; 1:500)] were performed as in (42). Three mice of each genotype and treatment were chosen for further analysis. Digital images of labeled islets were taken using the 20X objective of an Olympus BX41. Subjective assessments of islet anatomy were made using 3–4 representative islets from each mouse.

### Determination of Plasma Hormone Levels

For ghrelin, blood was collected into ice-cold EDTA-coated microfuge tubes. *P*-hydroxymercuribenzoic acid (final concentration 1 mmol/L; Sigma-Aldrich) was added, plasma was isolated following centrifugation, and HCl was added to achieve a final concentration of 0.1 nmol/L. For other hormones, blood was collected into 3 different EDTA-coated microfuge tubes. For glucagon, aprotinin (final concentration 250 KIU/ml; Sigma-Aldrich) was added. For catecholamines, EDTA-glutathione solution (9% w/v EDTA and 6% w/v glutathione, pH 7.4; 2 μL per 100 μL blood) was added. For insulin, no reagents were added.

ELISA kits were used for acyl-ghrelin (Millipore-Merck; Burlington, MA), insulin (Crystal Chem, Downers Grove, IL), and glucagon (Mercodia AB, Uppsala, Sweden). Calorimetric assays were performed using a BioTek PowerWave XS Microplate spectrophotometer (Winooski, VT) and BioTek KC4 junior software. Plasma catecholamines were determined using HPLC at the Vanderbilt University Medical Center Hormone Assay and Analytical Services Core.

### Statistical Analyses

All statistical analyses and graph preparations were performed using GraphPad Prism 7.0. A Student's *t*-test, 1-way, or 2-way ANOVA were used to test for significant differences among test groups. Data with significant unequal variance were log transformed prior to performing analyses. Outliers, if any, were detected by the ROUT test.

## Results

### STZ Induces Diabetes in Both WT and Ghrelin-KO Mice

**Figure 1:**
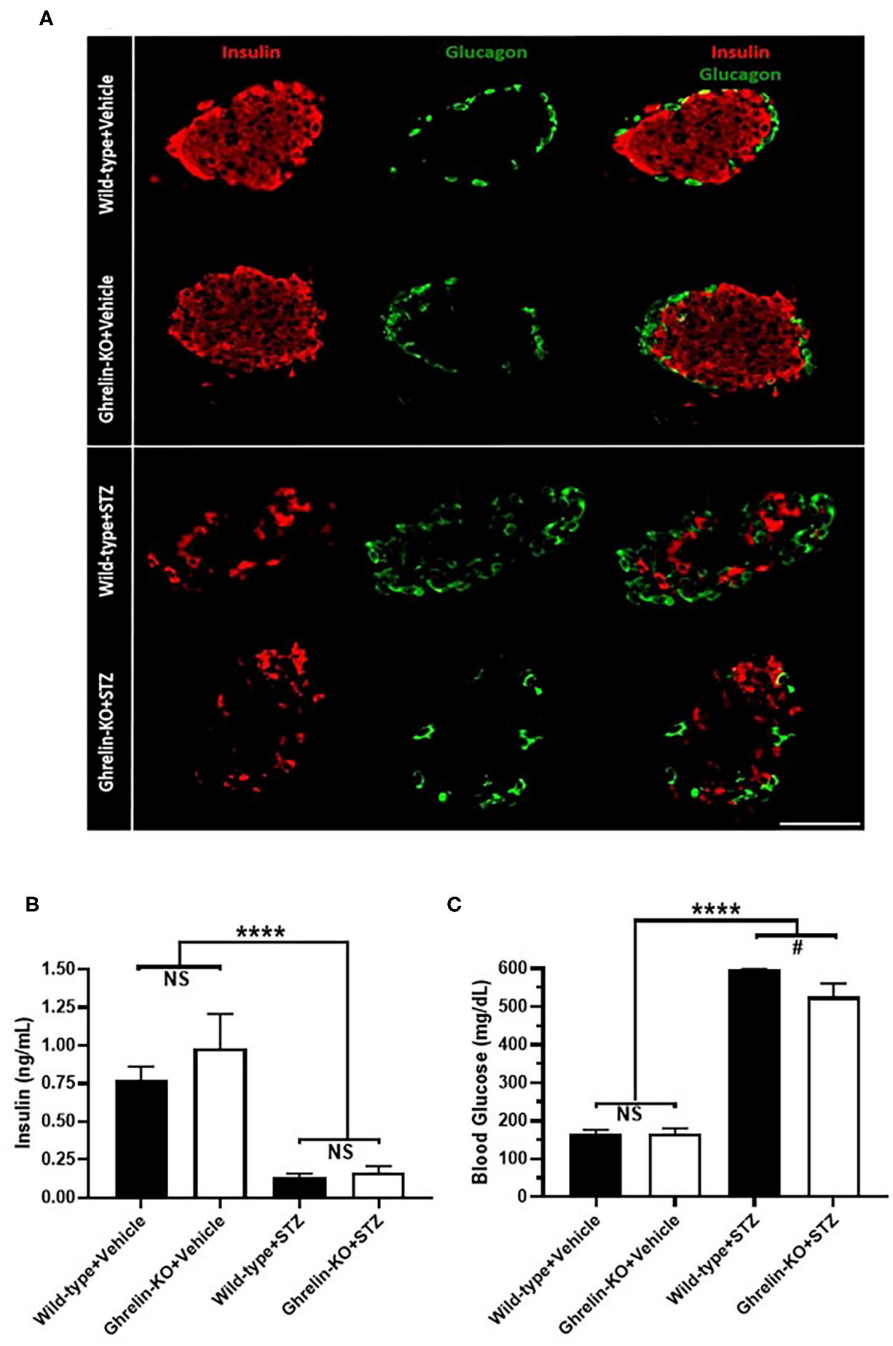
Effects of STZ on islets, plasma insulin, and blood glucose in WT and ghrelin-KO mice. **(A)** A representative islet co-labeled for insulin-immunoreactivity (red) and glucagon-immunoreactivity (green) from a vehicle-treated WT mouse (1st row), a vehicle-treated ghrelin-KO mouse (2nd row), an STZ-treated WT mouse (3rd row), and an STZ-treated ghrelin-KO mouse (4th row). Scale bar = 100 μm. **(B)** *Ad lib*-fed plasma insulin levels and **(C)** corresponding blood glucose levels in vehicle- and STZ-treated mice. Data are presented as mean ± SEM. *n* = 5–7. ^****^P < 0.0001, ^#^*P* = 0.09, NS, no significant difference.

A single high-dose of STZ (150 mg/Kg, i.p.) was administered to both ghrelin-KO mice and WT littermates in order to model type 1 diabetes mellitus. This administration protocol, which is based on one previously published (45–49) markedly reduced pancreatic β-cell mass (as assessed by comparing insulin-immunoreactivity in representative islets from vehicle-treated mice to insulin-immunoreactivity in islets from STZ-treated mice) in both genotypes without causing any subjective genotype-dependent differences in resulting islet shape, islet size, or patterns of insulin-immunoreactivity and glucagon-immunoreactivity (Figure 1A, Supplementary Figure 1). Plasma insulin levels also were equivalently reduced by STZ in WT and ghrelin-KO littermates (Figure 1B), while corresponding blood glucose levels were raised (Figure 1C). Thus, STZ efficaciously destroyed pancreatic β-cells in both WT and ghrelin-KO littermates—sufficiently enough to induce diabetes in both genotypes but, as expected (42, 49–52), without causing a complete disappearance of β-cells. Of interest, despite the induction of hyperglycemia in both genotypes, the mean blood glucose attained in ghrelin-KO mice was lower than that observed in WT mice (Figure 1C), similar to previously-reported effects of STZ in GHSR-null mice (42).

### STZ-Treated Ghrelin-KO Mice Are More Susceptible to Hypoglycemia During Hyperinsulinemic-Hypoglycemic Clamps

**Figure 2:**
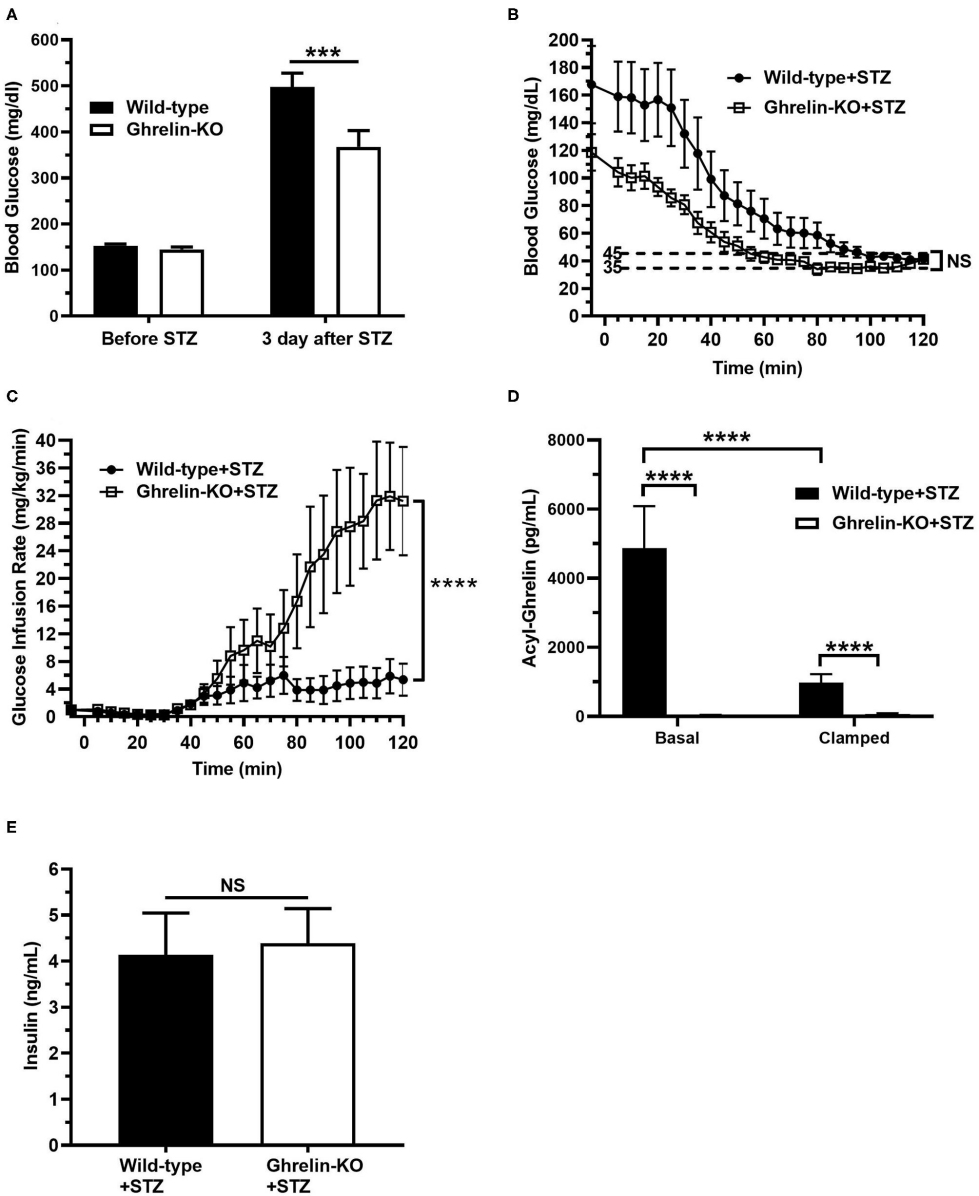
Low-dose hyperinsulinemic–hypoglycemic clamps in STZ-treated mice. **(A)** *Ad lib*-fed blood glucose levels before and 3 d after STZ administration. **(B)** Blood glucose levels during the clamps. **(C)** Glucose infusion rate (GIR) to achieve the target blood glucose (35–45 mg/dL) by the steady-state period (100–120 min) in mice receiving Humulin-R insulin (4 mU/kg/min) from 0 to 120 min. **(D)** Plasma ghrelin levels at the start (“Basal”; *t* = −5 min) and end (“Clamp”; *t* = 120 min) of the clamps. **(E)** Plasma insulin levels at the end (“Clamp”; *t* = 120 min) of the clamps. *n* = 10–11. Data are presented as mean ± SEM. ^***^P < 0.001, ^****^P < 0.0001, NS, no significant difference.

Next, we subjected a separate cohort of STZ-treated ghrelin-KO and WT littermates to a hyperinsulinemic-hypoglycemic protocol to assess the CRR. Just as shown in the above cohort (Figure 1C), STZ induced hyperglycemia in both WT and ghrelin-KO mice when assessed 3 d following STZ administration in the *ad lib*-fed state (Figure 2A). Also similar to the above cohort, the degree of hyperglycemia induced in ghrelin-KO mice was lower than that induced in their WT littermates (Figure 2A). Following a 16 h-fast to prepare the mice for the clamps, blood glucose levels fell in both genotypes, and remained lower in the ghrelin-KO mice than in the WT mice (Figure 2B). A low insulin dose protocol (4 mU/kg/min) was used, as in (41), to minimize direct inhibitory effects of insulin on ghrelin secretion in WT mice, while still achieving hypoglycemia. This low-dose insulin protocol reduced blood glucose in both STZ-treated WT and STZ-treated ghrelin-KO mice, such that within the final 20 min of the procedure, both genotypes were successfully clamped within the target hypoglycemic range (~35–45 mg/dL), with blood glucose levels that were without statistically significant differences (Figure 2B). Notably, a much higher (~5.8-fold) GIR was required by ghrelin-KO mice during the final 20 min of the clamps to maintain a similar blood glucose to that of WT littermates (Figure 2C).

We also determined plasma ghrelin and insulin levels. Basal plasma ghrelin in STZ-treated WT mice (Figure 2D) was significantly higher than levels usually observed in non-STZ-treated WT mice, as expected (34–41). Also as expected from other studies that examined the effects of insulin on plasma ghrelin or ghrelin secretion (41, 53–57), plasma ghrelin fell (~79%) in STZ-treated WT mice over the course of the clamp (Figure 2D). Nonetheless, plasma ghrelin remained much higher than the essentially undetectable level in ghrelin-KO littermates (Figure 2D). Also, plasma insulin levels were genotype-independent at the end of clamps, reflecting similar levels of endogenous plus infused insulin in the WT and ghrelin-KO mice (Figure 2E).

### The CRR Differs in STZ-Treated Ghrelin-KO and WT Littermates

**Figure 3:**
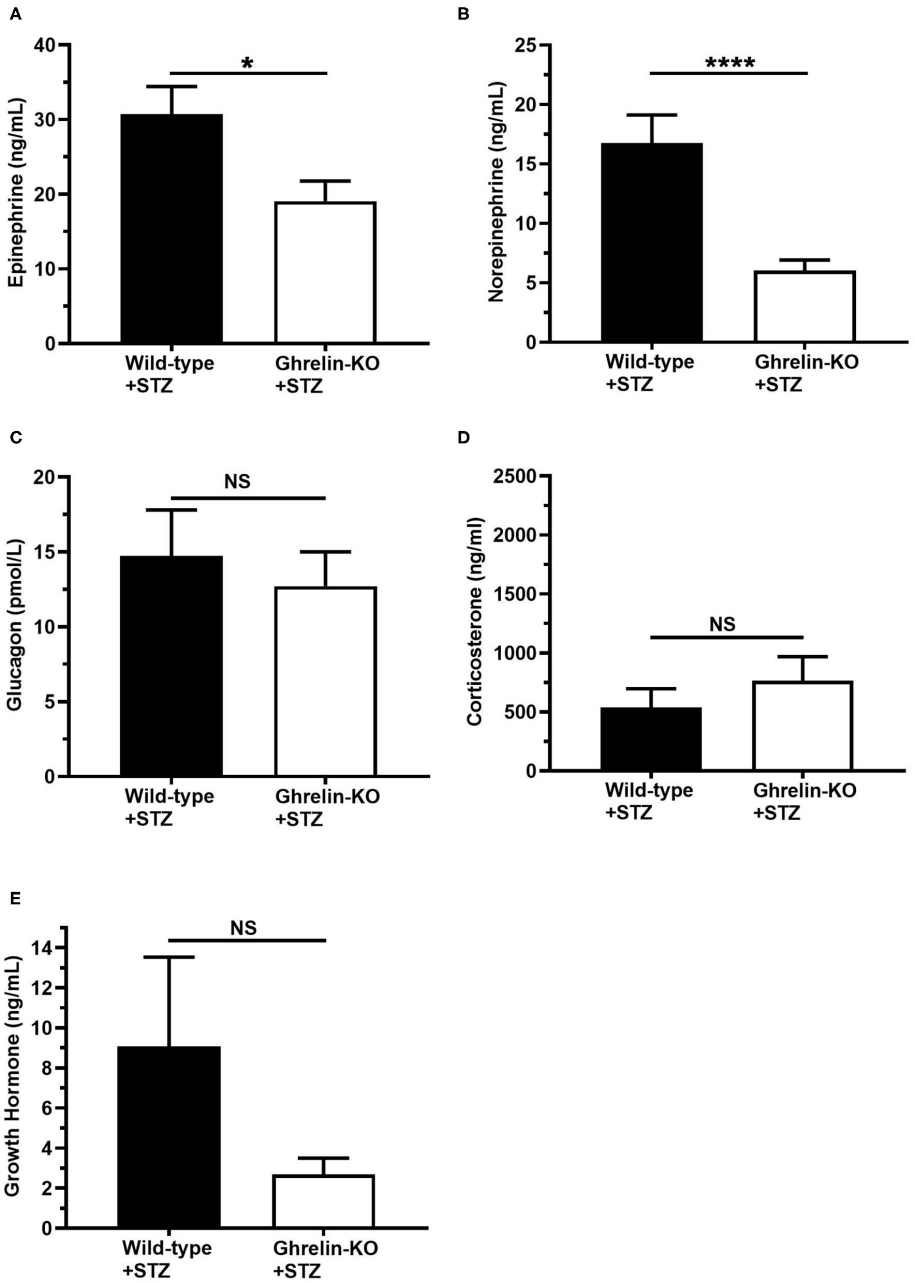
Effects of ghrelin deletion on plasma CRR hormone levels obtained at the end of the hyperinsulinemic-hypoglycemic clamps. Plasma levels of **(A)** epinephrine, **(B)** norepinephrine, **(C)** glucagon, **(D)** corticosterone, and **(E)** GH at the end of the clamps (*t* = 120 min). *n* = 10–11. Data are presented as mean ± SEM. ^*^P < 0.05, ^****^P < 0.0001, NS, no significant difference.

Finally, we assessed the levels of the traditional CRR hormones epinephrine, norepinephrine, glucagon, corticosterone, and GH at the end of the hyperinsulinemic-hypoglycemic clamps. Plasma epinephrine and norepinephrine were significantly lower in ghrelin-KO mice (Figures 3A,B). There were no genotype-dependent differences in end-of-clamp levels of plasma glucagon, corticosterone, and GH (Figures 3C–E).

## Discussion

The current study was designed to characterize the CRR actions of ghrelin during insulin-induced hypoglycemia in the setting of type 1 diabetes. Our new data indicate that ghrelin expression is permissive for the usual CRR to insulin-induced hypoglycemia in mice in which diabetes was induced by STZ. In particular, during hyperinsulinemic-hypoglycemic clamps, diabetic ghrelin-KO mice required a ~5.8-fold higher GIR to maintain blood glucoses in the target hypoglycemic range as compared to WT littermates. Previously, higher GIRs had been shown to be required by both non-diabetic ghrelin-KO and GHSR-KO mice as compared to WT mice during hyperinsulinemic-hypoglycemic clamps (41, 43). Interestingly, as compared to a previous study in which non-diabetic ghrelin-KO mice exhibited less robust corticosterone and GH responses during hyperinsulinemic-hypoglycemic clamps than WT littermates (41), the corticosterone and GH responses observed here in clamped diabetic ghrelin-KO and WT mice were without statistically significant differences. Instead, upon induction of hypoglycemia, the diabetic ghrelin-KO mice exhibited attenuated epinephrine and norepinephrine responses as compared to WT littermates.

There are several notable topics of discussion that these new data bring to mind. One relates to the demonstration of important actions by ghrelin during the CRR to insulin-induced hypoglycemia, now newly shown using the STZ model of type 1 diabetes. Just as had been observed in non-diabetic mice (41, 43), the elevated GIR required during the hyperinsulinemic-hypoglycemic clamps in STZ-treated ghrelin-KO mice as compared to WT littermates again suggests that the presence of circulating ghrelin is permissive for the usual CRR to insulin-induced hypoglycemia. This role for ghrelin during the CRR to insulin-induced hypoglycemia aligns with its other well-described glucoregulatory actions. These include effects of administered ghrelin and endogenous plasma ghrelin elevations to increase blood glucose (9, 10) and numerous examples of endogenously-produced ghrelin preventing falls in blood glucose—including life-threatening falls in blood glucose in certain settings, as reviewed in (10).

Our new data also highlight another observation previously made in insulin-induced hypoglycemic, non-diabetic mice—namely, that despite ghrelin expression allowing the usual CRR to insulin-induced hypoglycemia, plasma ghrelin levels do not elevate in STZ-treated WT mice upon hypoglycemia induction, as occurs with the traditional CRR hormones. Instead, plasma ghrelin levels fall over the course of the hyperinsulinemic-hypoglycemic clamps in the STZ-treated WT mice, just as occurs in non-STZ-treated mice (41). Nevertheless, despite this drop in plasma ghrelin, the remaining circulating ghrelin continues to serve a crucial permissive function during the CRR, as emphasized by the requirement for a markedly higher GIR and the attenuated changes to epinephrine and norepinephrine in clamped STZ-treated ghrelin-KO mice.

Another noteworthy topic of discussion relates to these lower epinephrine and norepinephrine levels. This finding suggests that the sympathoadrenal arms of CRR in the STZ-treated ghrelin-KO mice are blunted and potentially might contribute to their requirement for a higher GIR during the clamps. This blunted catecholamine response may result, at least in part, from decreased stimulation of hypothalamic corticotropin releasing factor (CRF) neurons. Notably, ghrelin induces c-fos expression and increases CRF expression in hypothalamic CRF neurons, induces CRF release from hypothalamic explants, and increases plasma ACTH and epinephrine in rodent and/or human subjects (58–63). Also relevant, epinephrine and norepinephrine are both potent ghrelin secretagogues, acting via ghrelin cell-expressed β_1_-adrenergic receptors to stimulate ghrelin secretion (30, 64). As mentioned, mice with ghrelin cell-selective deletion of β_1_-adrenergic receptors exhibit marked hypoglycemia when exposed to a week-long, severe caloric restriction regimen (30). Given their attenuated epinephrine and norepinephrine responses, hypoglycemic ghrelin-KO mice likely experience a reduction both in the effects that epinephrine and norepinephrine usually exert during hypoglycemia [namely, inhibition of whole body glucose utilization, increased delivery of gluconeogenic substrates to the liver, and enhanced hepatic and renal glucose production (65)] and in other protective glucoregulatory processes that would otherwise be initiated if epinephrine and norepinephrine could stimulate the release of more ghrelin [for instance, activation of brainstem or hypothalamic neurons, which in turn, raise blood glucose, as reviewed in (10)].

As mentioned, although the CRR response in ghrelin-KO mice during the hyperinsulinemic-hypoglycemic clamps had previously been shown to be attenuated in non-STZ-treated subjects (41), the deficits were different than those observed here. Specifically, attenuated plasma corticosterone and GH responses but normal epinephrine and norepinephrine responses had previously been observed in hypoglycemia-clamped non-STZ-treated ghrelin-KO mice (41). These contrast with the observations here of attenuated plasma epinephrine and norepinephrine responses but normal corticosterone and GH responses in hypoglycemia-clamped STZ-treated ghrelin-KO mice. These different patterns of traditional CRR hormone responses in clamped ghrelin-KO mice treated with STZ vs. non-STZ-treated animals might result from one or more of the known differential CRR responses in individuals with and without diabetes. For instance, loss of functional β-cells in the diabetic islet causes α-cell dysregulation, and in turn, loss of normal tonic inhibition of α-cells by intra-islet insulin (8). Also, mRNA levels of adrenal tyrosine hydroxylase, which is the rate-limiting enzyme in catecholamine biosynthesis, are decreased in STZ-induced diabetic rats (66). Interestingly, the observed catecholamine deficits observed here in the clamped STZ-treated ghrelin-KO mice are reminiscent of the attenuated sympathoadrenal system response associated with diabetes in the form of hypoglycemia-associated autonomic dysfunction (5). More studies are needed to determine if reconstitution of the epinephrine and norepinephrine responses in STZ-treated ghrelin-KO mice could rescue their deficient CRR, and also to determine if an aberrant ghrelin response might contribute to the occurrence of hypoglycemia-associated autonomic dysfunction.

There are some caveats to our study that should be kept in mind when interpreting the results. First, the STZ mouse model adapted for the current study from several studies in the literature (45–49) does not completely mimic the pathophysiology or presentation of type 1 diabetes in humans. For instance, although STZ is toxic to pancreatic β-cells, reducing their numbers enough to lower circulating plasma insulin and raise blood glucose into the hyperglycemic range, some β-cells remain (Figure 1A, Supplementary Figure 1) (67), unlike that observed in humans with long-standing type 1 diabetes. Although plasma ghrelin elevates upon STZ administration (Figure 2D) (36, 38, 40, 42) and also although this elevated ghrelin is thought to contribute to STZ-induced hyperphagia (38), changes to plasma ghrelin have not been consistently reported in the literature in type 1 diabetes in humans (33, 68–73) nor is hyperphagia a usual feature of type 1 diabetes in humans. Also, following STZ induction of diabetes, the mice did not receive insulin to treat the hyperglycemia until the clamp procedure, which is unlike the optimal situation in humans with type 1 diabetes in which normoglycemia would be the goal. Additionally, these mice have new-onset diabetes as opposed to most individuals with type 1 diabetes who have been living with the disorder for years. Nonetheless, STZ as administered here reduced plasma insulin substantially (by 82–84%), via efficacious reduction in pancreatic β cell mass, and induced hyperglycemia, which are key hallmarks of type 1 diabetes.

A second limitation of the study relates to the potential influence of the collection method for the blood samples used to measure epinephrine and norepinephrine. Specifically, the samples for CRR hormones were collected by cardiac puncture from isoflurane-anesthetized mice at the end of the clamps. Although handling of the mice was minimized during the clamps, frequent sampling of blood from tail nicks to assess blood glucose was performed throughout the 2 h clamp procedure. Prior work has shown that blood sampling from tail nicks as compared to indwelling arterial cannulas induces a rise in plasma catecholamines (74). Thus, the relatively high levels of catecholamines observed here [in the ~20–30 ng/mL range for epinephrine and the ~5–17 ng/mL range for norepinephrine] as compared to those in another hypoglycemic clamp study (74) [in the ~0–1.2 ng/mL range for epinephrine and the ~0.21–0.42 ng/mL range for norepinphrine], in which blood sampling from non-STZ-treated mice was performed via indwelling arterial cannulas, may have been influenced by the blood collection methods. Other potential stressors, including the recent STZ treatment, the days-long period of hyperglycemia, the 16 h fast preceding the induction of hypoglycemia, and background genetic strain of the mice also likely impacted the magnitude of the detected catecholamine levels (41, 75). That said, ghrelin-KO and WT mice were exposed to the same stressors and were handled exactly the same during the study, and yet, epinephrine and norepinephrine levels were lower in the ghrelin-KO mice. Thus, we predict the differences observed in catecholamine levels between ghrelin-KO and wild-type littermates reflect a genotype-dependent, differential response to hypoglycemia.

A third caveat relates to the use of ghrelin-KO mice, which, because of long-term absence of ghrelin, could exhibit a phenotype reflective of potential compensatory developmental changes that alter the true effects of absent ghrelin action in the setting of insulin-induced hypoglycemia. That said, at least when assessed in non-STZ-treated subjects, ghrelin receptor agonist administration has been shown to rescue the deficits observed in ghrelin-KO mice under the hyperinsulinemic-hypoglycemic protocol (41).

## Materials Availability

All unique reagents, mouse lines generated in this study will be made available on request, but we may require a payment and/or a completed Materials Transfer Agreement if there is potential for commercial application.

## Data Availability Statement

All datasets generated for this study are included in the article/Supplementary Material.

## Ethics Statement

The animal study was reviewed and approved by UT Southwestern Medical Center Institutional Animal Care and Use Committee.

## Author Contributions

KS conceptualized and performed the experiments, analyzed and interpreted the data, and helped write the manuscript. BF, SO-L, and NM performed the experiments. DG performed the experiments and helped analyze and interpret the data. BM conceptualized and performed some experiments. CL helped design and generate the ghrelin-KO mice. EB conceptualized the experiments, secured funding, interpreted the data, and supervised the research activity. JZ conceptualized the experiments, secured funding, interpreted the data, supervised the research activity, and helped write the manuscript. All authors contributed to the article and approved the submitted version.

## Conflict of Interest

The authors declare that the research was conducted in the absence of any commercial or financial relationships that could be construed as a potential conflict of interest.
