# Supplementary material for: Ghrelin Protects Against Insulin-Induced Hypoglycemia in a Mouse Model of Type 1 Diabetes Mellitus
Source: Front Endocrinol (Lausanne). 2020 Sep 11;11:606. doi: 10.3389/fendo.2020.00606 (PMC7518392; doi:10.3389/fendo.2020.00606)
Supplement: Supplementary file 1 [file Data_Sheet_2.PDF]

Supplementary Figure 1.

Islet histology of STZ-treated Wild-type and ghrelin-KO littermates. Ten representative islets from 3 STZ-treated wild-type mice (A) and from 3 STZ-treated ghrelin-KO littermates (B). Notably, 10 islets from each mouse were visualized, and 3-4 representative islets from each mouse were included in the analysis. Each islet is co-labelled for insulin-immunoreactivity (red) [guinea pig anti-Insulin (DakoCytomation, Carpinteria, CA; diluted 1:300) followed by Alexa Fluor 594® donkey anti-guinea pig IgG (ThermoFisher Scientific; 1:500)] and glucagon-immunoreactivity (green) [rabbit anti-Glucagon (Millipore, Temecula, CA; diluted 1:300) followed by Alexa Fluor 488® donkey antirabbit IgG antibody (ThermoFisher Scientific; 1:500). Scale bars =100 μm.

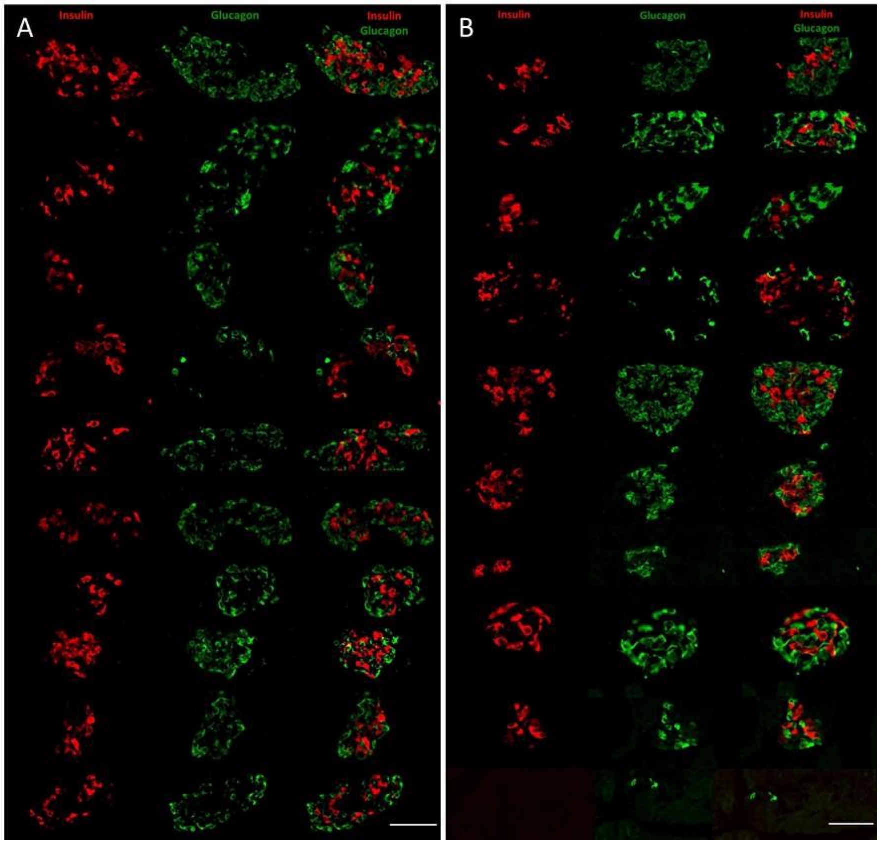

Supplementary Figure 1
